# Investigating weighted fishing hooks for seabird bycatch mitigation

**DOI:** 10.1038/s41598-022-06875-4

**Published:** 2022-02-18

**Authors:** Eric Gilman, Michael Musyl, Michael Wild, Hua Rong, Milani Chaloupka

**Affiliations:** 1 The Safina Center, Honolulu, HI USA; 2 Heriot-Watt University, Edinburgh, UK; 3 Pelagic Research Group, Honolulu, HI USA; 4 MGM Fisheries, Honolulu, HI USA; 5 Jessn Marine Equipment, Ningbo, China; 6 Ecological Modelling Services Pty Ltd & Marine Spatial Ecology Lab, University of Queensland, St. Lucia, QLD Australia

**Keywords:** Ecology, Ecology, Environmental sciences, Environmental impact

## Abstract

Fisheries bycatch threatens the viability of some seabird populations and reduces fishing efficiency. Albatross bycatch in a US North Pacific tuna longline fishery has increased over the past decade and now exceeds 1000 annual captures. Seabirds interacting with this fishery reach hooks at depths up to 1 m. A branchline weight’s mass and distance from the hook affect seabird catch rates. We conducted experimental fishing to compare the commercial viability of a weighted hook relative to conventional gear with weights attached 0.75 m from the hook. We used a Bayesian random effects meta-analytic regression modelling approach to estimate pooled expected species-specific log relative risk of capture on conventional versus experimental gear. There was a significant 53% (95% HDI: − 75 to − 25%) decrease in retained species’ catch rates on experimental hooks, indicating an unacceptable economic cost, and no significant effect for discarded species. Using a Bayesian general linear mixed regression modelling approach, experimental hooks sank to 85 cm *ca*. 1.4 times (95% HDI: 1.37–1.48) faster than control hooks. Given their potential to reduce seabird catch rates, eliminate safety risks from bite-offs and facilitate robust compliance monitoring, it is a priority to find a weighted hook design with acceptable catch rates.

## Introduction

Bycatch in longline and other fishing gear types is a serious threat to albatrosses and petrels, which are two of the three most threatened groups of seabirds^1–3^. Bait loss to scavenging seabirds and handling caught seabirds reduces fishing efficiency^4–6^. Over 90% of the seabird bycatch in the US central North Pacific tuna longline fishery is comprised of Laysan (*Phoebastria immutabilis*) and black-footed (*P. nigripes*) albatrosses, which are categorized as Near Threatened with stable and increasing population trends, respectively^3,7^. Following the introduction of seabird regulations in the fishery, there was a 67% decline in standardized seabird catch rates^8^. However, seabird catch levels have been significantly increasing over the past decade due to increasing fishing effort and increasing black-footed albatross catch rates^7,9^. The increasing catch rate is attributable to an increase in the number of black-footed albatrosses attending vessels, possibly in response to changes in seasonal and spatial distribution of fishing effort, variability in ocean productivity linked to inter-annual and decadal climate cycles as well as to outcomes of climate change, and to the increasing population trend of black-footed albatrosses^7,10^. The most recent seabird bycatch estimate for the fishery, for 2019, was 1002 captures, of which 96% were retrieved dead, and likely were captured during setting^7,11^.

Numerous gear technology methods have been demonstrated to reduce seabird bycatch that are also commercially viable and facilitate compliance monitoring in pelagic longline fisheries, including branchline weighting designs^5,12,13^. Required seabird bycatch mitigation measures for this US longline fishery include the attachment of a weight of ≥ 45 g within 1 m of the hook^14^. The mass of a pelagic longline branchline’s weight and distance between the weight and the hook significantly affect seabird catch risk during setting^15–18^, as well as during the gear soak and haul^19,20^. During gear setting, these two variables affect the sink rate of baited hooks and their availability to seabirds. The closer a weight is to the hook, the less time it takes for the weight to affect the hook’s sink rate near the surface and the less time baited hooks are available to surface-foraging seabirds^15,21^. Similarly, during the gear soak (when catch may bring the gear to the surface, and when the gear shoals from currents and wind) and haul (when hooks can drag near or at the surface as crew coil branchlines), the closer a weight is to the hook, the more likely the weight will reduce the availability of hooks to surface-foraging seabirds^19^.

All three genera of small albatrosses (*Phoebastria*, *Thalassarche* and *Phoebastria*) predominantly make infrequent and short dives to a mean depth of only 1.3 to 1.5 m, and only infrequently will make prolonged, deeper dives^22,70^. Laysan and black-footed albatrosses in particular have relatively limited diving capacities^23^, typically only making body thrusts to reach prey near the surface. Black-footed albatrosses make dives to a mean depth of 0.6 m and have a maximum diving depth of about 2.5 m^24^. Furthermore, unlike in other regions, secondary interactions are understood to not occur in North Pacific pelagic longline fisheries ^7^. Secondary interactions occur when deep-diving seabirds access baited hooks at depth and return them to the sea surface providing larger surface-foraging seabird species with a second opportunity to access the terminal tackle and become captured^17,25^.

Seabird catch risk in the US longline fishery is therefore probably highest at the moment baited hooks contact the sea surface during setting, and when crew bring baited hooks close to the surface during the haul^19^. In this fishery, the baited hook’s sink rate over the initial *ca.* 1 m of the surface is likely where seabird catch risk occurs, and not to 10 m, which has been used for assessments of the effect of baited hook sink rate on the catch risk of *Procellaria* petrels in studies of southern hemisphere longline fisheries^26^. These observations support the hypothesis that placing weights at the hook instead of the predominant design in Hawaii’s tuna longline fishery of attaching 45 g weighted swivels 0.7 m from the hook, might reduce Laysan and black-footed albatross catch risk.

In mid-2021, the fishery transitioned from using wire to monofilament leaders in order to reduce shark catch rates, in response to a recent listing of the oceanic whitetip shark (*Carcharhinus longimanus*) as threatened under US law^27,28^. With this change in leader material, the safety risk to crew from flybacks resulting from bite-offs of terminal tackle increased^19,29–31^. Relocating branchlines weights to the hook could reduce this source of safety risk, as the hook and weight would now be bitten off.

We conducted experimental fishing to compare the commercial viability of a weighted longline hook relative to conventional gear with weights located 0.75 m from the hook. Following the experimental fishing, we obtained the fishers’ perspectives on safety, practicality and economic viability. We also conducted a mechanistic study to compare the hook sink rates of experimental and control branchlines. The study explored the effects on commercial viability and sink rate of a new method to mitigate seabird bycatch that is relatively safe and practical, and facilitates effective compliance monitoring even in fisheries with rudimentary surveillance frameworks.

## Methods

The U.S. Fish and Wildlife Service was the institutional committee that approved the experimental protocols and issued a determination that the project met all environmental compliance requirements (reference number 01EPIF00-2021-TA-0052). All experiments were performed in accordance with relevant guidelines and regulations. The reporting in the article follows the recommendations in the Animal Research Reporting of In Vivo Experiments (ARRIVE) guidelines.

### Experimental and control treatments

**Figure 1:**
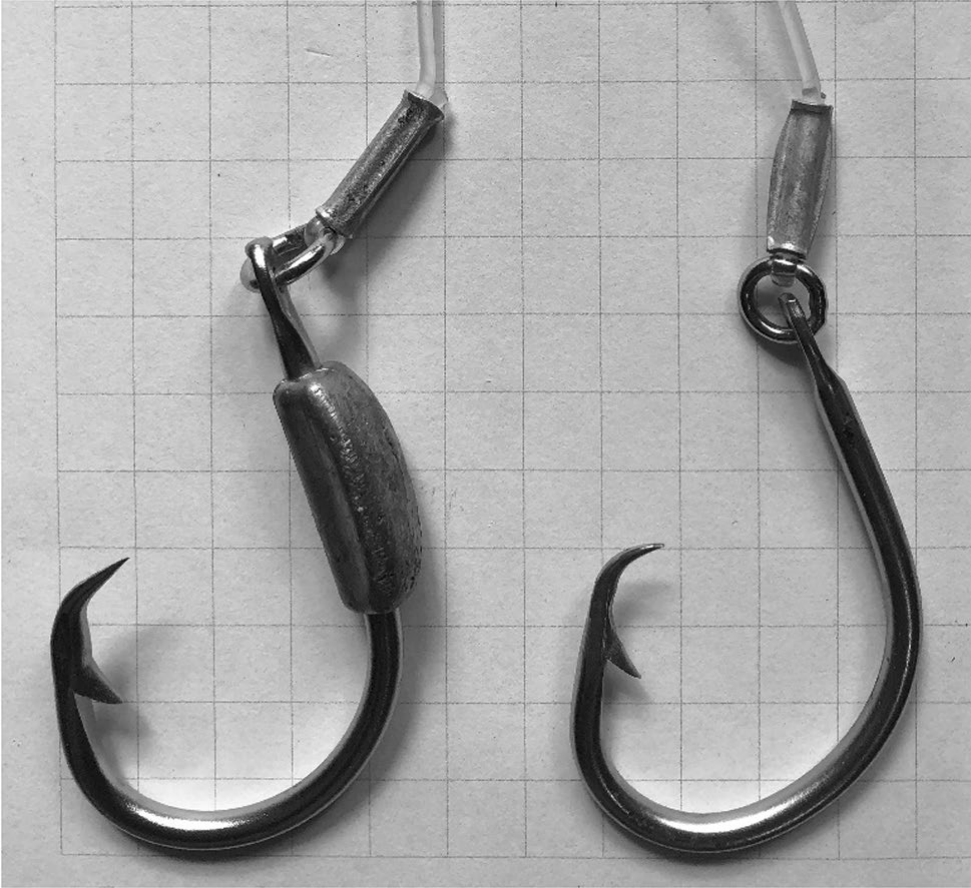
Control and experimental hooks. Squares have 1 cm length sides.

Control branchlines used the vessel’s conventional gear design with 45 g lead-centered swivels attached to the branchline a mean of 74.7 cm (± 6.9 SD) from the eye of the hook. Branchlines and leaders were 2.0 mm clear monofilament nylon. Control hooks were 4.5 mm diameter round wire, 42.0 mm minimum width, 21.5 mm gape, 10° offset circle hooks with a ring. The experimental weighted hooks had the same wire diameter, offset, shape and ring, but had a wider 43.5 mm minimum width. The larger minimum width allowed the experimental hook with lead with a mass of 32 g added to the shank to maintain the same gape as the control hook (Fig. 1). Both hooks had a mass of 14 g, not including the mass of the lead added to the experimental hook. Pacific saury (*Cololabis saira*) was used as bait in both treatments.

### Sink rate

We used an adapted ‘bottle test’ method^32–35^ to measure the sink rate of experimental and control treatment hooks (Supplementary Fig. S1). A 0.25 mm diameter, 82 cm long, 3.6 kg (8 lb.) test nylon monofilament fishing line (hereafter referred to as the sink rate line) was tied to the eye of the hook, and the other end of the line was connected to the neck of an empty, sealed 473 ml (1 US pint) plastic bottle filled half-way with water for ballast. For the tests, the branchline and sink rate line were held horizontally (i.e., parallel to the sea surface) and dropped into the water to approximate the orientation of the terminal tackle of a conventional branchline when set by crew^36^. The study was conducted at a location with static environmental conditions (no wind, waves or current). We did not include bait to avoid confounding effects of the variability in degree of thawing and physical characteristics (e.g., size and weight) between individual baits. The control branchline used in the sink rate assessment had a 69.0 cm leader length (bottom of the swivel to eye of the hook) (Supplementary Fig. S1).

A power analysis using a two-tailed *t-*test on the difference between means of the two treatments with α = 0.05 and β = 0.10 (i.e., 90% power in the experiment or the chance of detecting a real effect) indicated a sample size of 44 paired tests would provide a 95% confidence interval of the effect size to within ± 0.30 points. A median effect size of 0.5 m/s was employed based on data from Barrington et al.^26^.

Fifty-one replicates were conducted per treatment, with treatments deployed in pairs, where 1 control and 1 experimental branchline were simultaneously recorded for sink time. Wondershare Filmora X video editor software^37^ was used to measure, to the nearest 0.1 s, the elapsed time between the hook contacting the surface and the bottle turning to a fully vertical position at the surface, which occurred when the lower 3 cm of the bottle was submerged, and thus when the hook was at 85 cm depth.

The statistical modelling approach was based on a Bayesian inference workflow^38,39^ applying a general linear mixed regression model (GLMM) with Gaussian likelihood^40^ to determine if the mean sink time was dependent on whether experimental or control hooks were used in the 51 paired trials. The response variable was the time in seconds for the hook to sink to 85 cm. The model was fit using the Stan computation engine^41^ via the brms interface^42^. See the Supplemental Material for more details on the statistical modeling approach.

### At-sea demonstration and experiment

A controlled experiment was conducted on the Hawaii-based F/V Kilauea during one trip of 13 sets made between 30 July and 11 August 2021. There were 3600 hooks per set, with 25 hooks between two floats. For general information on this fishery, including fishing methods and gear designs, see Refs.^7,8,11,69^. The experimental design was balanced with 1800 branchlines of each of the two treatments deployed per set. Cable ties were attached to branchline clips to identify which treatment was used for each branchline, in case the terminal tackle was lost and needed to be replaced. During the first set, treatments were alternated every branchline, and then the experimental and control treatments were allowed to randomly mix during subsequent sets. At the end of each set, any damaged or lost branchlines were replaced with the same treatment in order to maintain the same number of control and experimental branchlines in each set. At the end of the fishing trip, the captain and crew were interviewed to document their perspectives on the practicality, safety and economic viability of the experimental weighted hook.

The following information was recorded on the catch: (1) species, (2) treatment it was captured on, and (3) whether it was retained or discarded. At the start of each set, the order of hook type for the first 100 hooks stored in one bin was recorded. Following the methods of Gilman et al.^43^, we used the DescTools package for R^44^ to perform set-specific Wald–Wolfowitz test for runs^45^ for each sampled hook order series by set in order to test the hypothesis of randomness, that there was no significant difference between the number (size classes) of runs of each of the two hook types after the first set.

A random-effects meta-analytical modelling approach^46^ was used to estimate log relative risk of capture on a control branchline or an experimental branchline using catch-effort data from the 13 sets for each of 17 captured species. Specifically, the species-specific log relative risk ratio and variance were derived using the escalc() function in the metafor package for R^47^. Then, an inverse-precision weighted Bayesian regression model with Gaussian likelihood was fitted to those effect sizes to estimate the overall log relative risk of capture with species as the random effect^48^. We fitted separate models to the retained and discarded species data using the Stan computation back-end^41^ via the brms interface for R^42^ with weakly informative priors ^49^.

The model-specific results were then summarized in a forest plot of the species-specific mean posterior densities to display the model-predicted mean posterior estimates and 95% highest posterior density intervals (HDIs)^50^. The full posterior distribution for each estimate is also shown to support a more precise form of communicating parameter uncertainty^51^. The ggplot2^52^ and colorspace^53^ packages for R were used for all summary graphics. We used the back-transformed log relative risk posterior samples to calculate the percent reduction in catch risk for experimental branchlines using the HDI as the measure of uncertainty (see Gilman et al.^31^ for details).

## Results

### Sink rate

**Figure 2:**
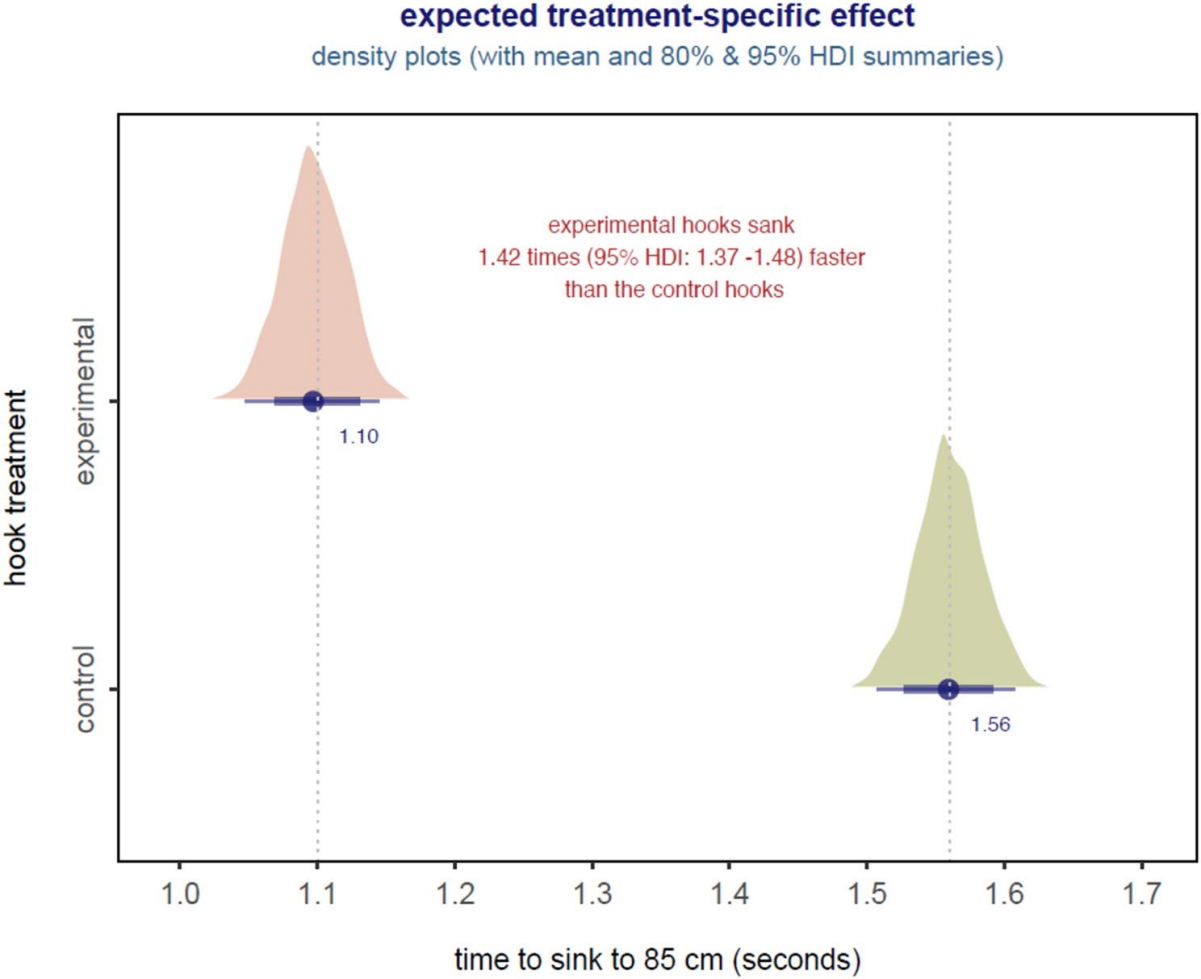
Summary of the estimated hook treatment effect derived from the GLMM for the 51 paired sink rate trails. Colored polygon shows the density distribution summary for that parameter, solid dot (+ numeric label) = estimated mean of the density polygon, thick horizontal line below each polygon shows the 80% HDI for the posterior density polygon while the thin horizontal line is the 95% HDI.

The Bayesian GLMM with Gaussian likelihood was a good fit to the sink elapsed time data (Supplementary Fig. S2) and all diagnostics reflected model convergence. All inference was based on this model. Experimental hooks sank to 85 cm significantly faster than did control hooks (Fig. 1). The expected or mean sink elapsed time for experimental hooks was about 1.1 s (95% HDI: 1.05–1.15) while the mean sink rate for controls hooks was about 1.6 s (95% HDI: 1.51–1.61). Experimental hooks sank an estimated 1.42 times (95% HDI: 1.37–1.48) faster than control hooks (Fig. 2).

### Random order

The Wald-Wolfowitz test for runs found that 69% of 13 sets did not have a significantly different number of runs of the two hook types, suggesting that they were in randomized order. In the remaining 4 sets, there were significantly more runs of one hook size than expected. There was no evidence that hooks became increasingly or decreasingly nonrandomly ordered as the sets proceeded within the trip, suggesting that there was no systematic process biasing the order of hooks. The 31% of sets that showed significant nonrandom hook order were probably due to chance. A robust risk aggregation metric using an adjusted geometric mean^54^ was used here to combine all 13 Wald-Wolfowitz test *p*-values into a single *p*-value, which was *p* = 0.204—suggesting that overall, the study used adequately randomized assignment of hook types across the 13 sets.

### Catch rate effect

During the experiment, 383 fish were captured. No seabirds, or other non-fish species (marine turtles, marine mammals), were captured. All but 1 captured tunas (N = 143), all wahoo (*Acanthocybium solandri*, N = 30), all mahi mahi (*Coryphaena hippurus*; N = 23), all but 1 combined escolar (*Lepidocybium flavobrunneum*) and oilfish (*Ruvettus pretiosus*) (N = 16), all billfishes (N = 11), all monchong (*Taractichthys steindachneri*, N = 8), and all opah (*Lampris* spp., N = 3) were retained. We refer to these as retained species. Fishers discarded all lancetfishes (*Alepisauridae* spp., N = 104), blue sharks (*Prionace glauca*, N = 33), thresher sharks (*Alopias* spp., N = 10), and snake mackerel (*Gempylus serpens*, N = 2), and we refer to these as discarded species.

**Figure 3:**
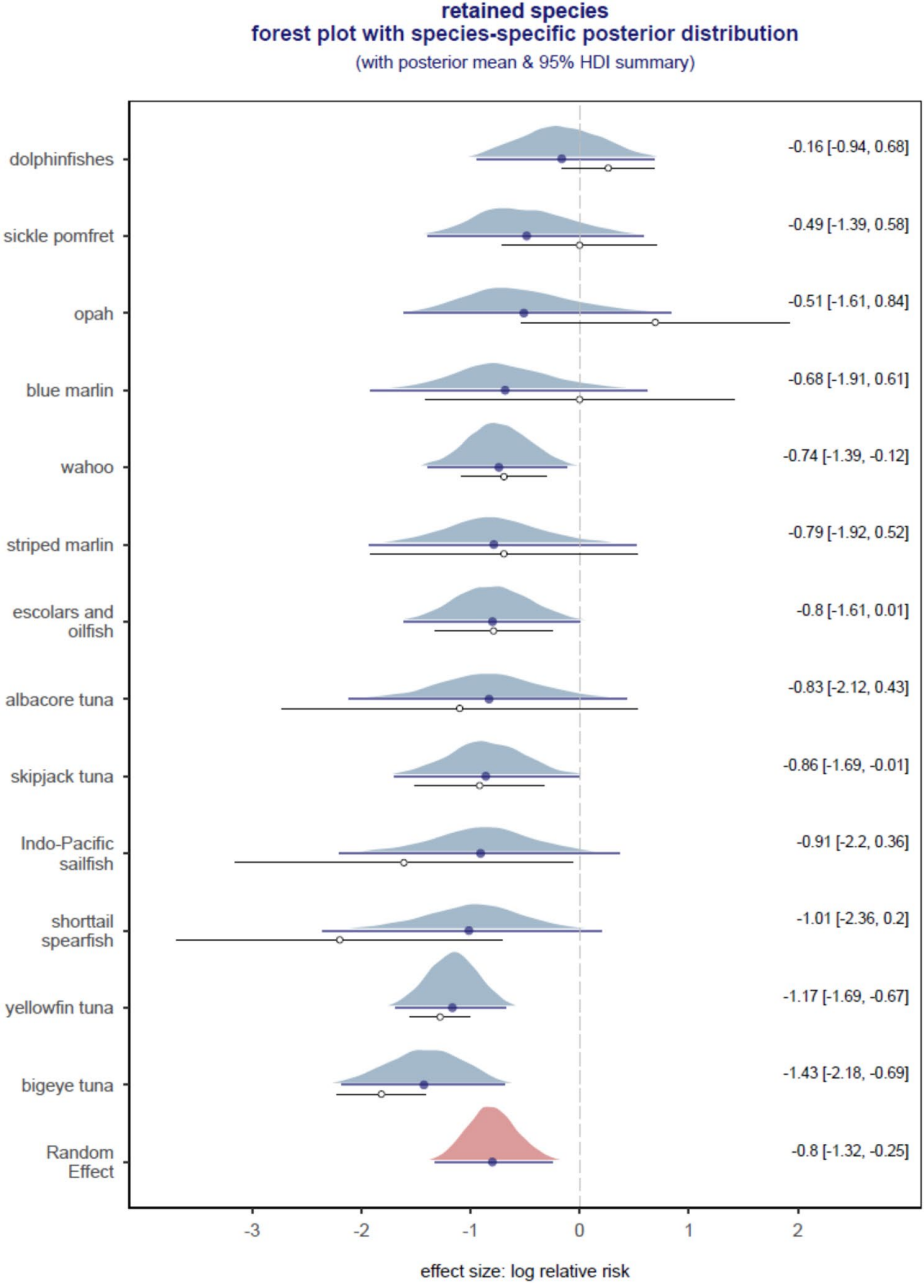
Model-predicted log relative risk ratio derived for 13 species-specific effect sizes for retained species. Shrinkage estimates were derived using a precision weighted Bayesian random-effects meta-analytic model with Gaussian likelihood. Polygon = density of the posterior draws (the effective sample size = 10,000) and the horizontal line underneath each polygon = 95% highest posterior density interval (HDI) of the posterior draws, solid dot = mean of the posterior draws shrunk towards the pooled or Random Effect, which is the mean or overall expected log relative risk ratio for all 13 species (right-side labels = the posterior mean and HDI summaries). Below the density polygon is an open dot = observed effect size and thin horizontal line = observed effect size ± 1 standard deviation derived using the metafor::escalc() function. The difference between solid and open dots reflects the degree of shrinkage that is dependent on sample size.

**Figure 4:**
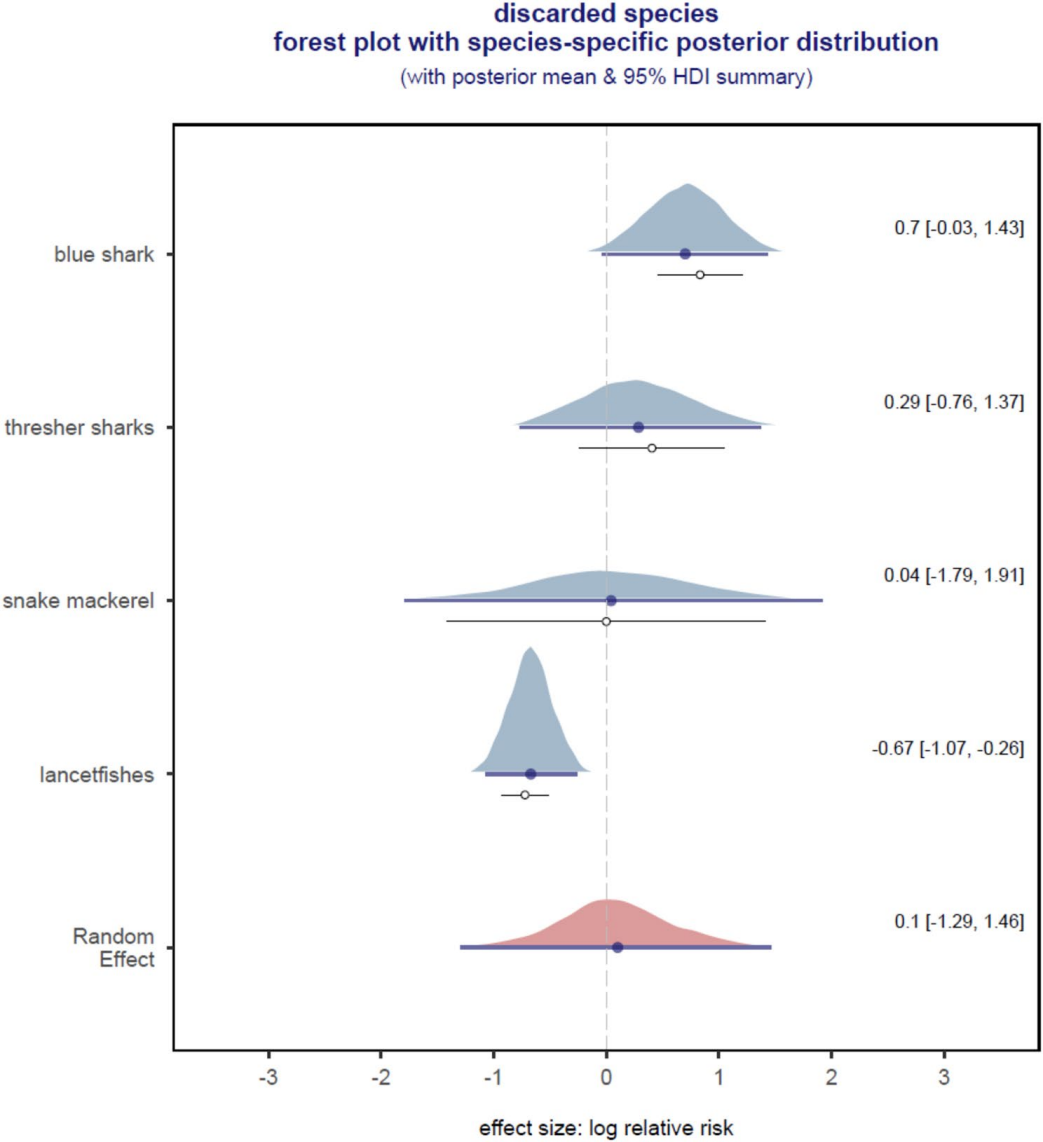
Model-predicted log relative risk ratio derived for 4 species-specific effect sizes for discarded species. Shrinkage estimates were derived using a precision weighted Bayesian random-effects meta-analytic model with Gaussian likelihood. Polygon = density of the posterior draws (the effective sample size = 10,000) and the horizontal line underneath each polygon = 95% highest posterior density interval (HDI) of the posterior draws, solid dot = mean of the posterior draws shrunk towards the Random Effect, which is the overall expected log relative risk ratio for all 4 species (right-side labels = the posterior mean and HDI summaries). Below the density polygon is an open dot = observed effect size and thin horizontal line = observed effect size ± 1 standard deviation derived using the metafor::escalc() function. The difference between solid and open dots reflects the degree of shrinkage that is dependent on sample size.

Figures 3 and 4 are forest plots summarizing the model-predicted log relative risk ratios and the estimated pooled random effects for retained and discarded species, respectively. Species-specific weighted relative risk estimates are ordered by effect size, and an estimated overall pooled or Random Effect log relative risk ratio is shown at the bottom of the forest plots. A log relative risk > 0 (to the right of the dashed vertical line) indicates a higher relative risk of capture on experimental branchlines with weighted hooks than control branchlines, and 0 indicates no branchline-specific effect on catch rates. The blue shaded polygon is the density plot of model simulations and reflects the distribution of each estimate, where a wide and thin polygon indicates low precision from a small sample size, and narrow and tall indicates there was high precision and large sample size. The solid dot is the posterior mean shrunk towards the Random Effect, and thick horizontal bars are 95% HDIs. The open dot below the density polygon is the observed effect size, and thin horizontal line is the observed effect size ± 1 standard deviation. The difference between solid and open dots reflects the degree of shrinkage that is dependent on sample size.

The overall random effect estimate of relative risk was significant for retained species but not for discarded species. There was a 53% (95% HDI: − 75% to − 25%) decrease in retained species catch rates on experimental branchlines and we can be > 99% certain that the overall average or random effect shown in Fig. 3 was < 0. No significant effect was found for the discarded species catch rates with the posterior probability of any effect being only around 56% (Fig. 4). For the 4 discard species, catch rates on the weighted hook were significantly higher for blue shark, significantly lower for lancetfishes, and not significantly different for thresher shark and snake mackerel (Fig. 4).

### Fisher perspectives on commercial viability and safety

According to the captain and crew, building branchlines with the weighted hook was slightly easier than the convention design. The captain and deck boss were asked to design the experimental branchlines if they were using the weighted hook under normal commercial fishing conditions, and they chose not to include a swivel to replace the conventionally-used 45 g lead-centered swivel. This resulted in one fewer element to incorporate into the terminal tackle.

The same number of branchlines with weighted and conventional hooks were able to be stored in each bin, and there was no noticeable difference between the branchlines in rates of tangling in the bins. The weighted hook did not affect gear setting, including the ability to thread a bait onto the hook nor the hook setting speed. However, when hauling, likely because no swivel was near the terminal end of the experimental branchline, the line occasionally became twisted and tangled, especially when a bait was on the hook, which crew had to untangle before they could coil and store the branchlines in bins.

There were only two flybacks during the trip, one each on the experimental and conventional hooks, and therefore the weighted hook had no apparent effect on the rate of flybacks and crew safety. There were about equal rates of catch severing the line or branchlines breaking on the two branchline designs. However, there was a higher rate of fish throwing (the hook dislodges or is torn out of the catch) weighted hooks than conventional hooks. The crew observed that when catch would thrash and struggle when they were retrieving a branchline, the weighted hook created a wound, which may have caused a higher escapement rate from catch throwing the hook.

## Discussion

### Sink rate

Experimental hooks sank about 1.4 times faster than control hooks, reaching 85 cm, which is roughly the estimated typical diving depth of black-footed and Laysan albatrosses^23,24^, on average in about 1.1 s while control hooks reached this depth in about 1.6 s. For a vessel with a setting speed of 7 knots (3.6 m/s) with negligible inline current, by time conventional and experimental hooks reach 0.85 m, the vessel would have moved about 5.8 m and 4.0 m, respectively, from the point where the baited hooks contacted the surface. Seabirds may be unwilling or unable to pursue baited hooks that are near the vessel^55^. This faster sink rate and shorter distance astern from the vessel for weighted hooks to reach 0.85 m relative to control hooks could potentially cause a large reduction in seabird catch risk for surface-scavenging seabirds, including Laysan, black-footed and other small albatrosses, which make relatively shallow dives^22–24^. The sink rates estimated here (0.77 m/s and 0.53 m/s, experimental and control hooks, respectively) both exceed the Agreement on the Conservation of Albatrosses and Petrels’ best practice minimum sink rate of 0.3 m/s^13^.

Robertson et al.^15^ conducted a comparable sink rate comparison with similar results. Estimating elapsed time using time-depth recorders (TDRs) set to record every second, with 30 replicates per treatment, Robertson et al.^15^ estimated that, in static water, baited hooks with 60 g weights attached 1 m from the hook and immediately adjacent to the hook reached 2 m at mean elapsed times of 3.53 s and 2.63 s, respectively, but the difference was not statistically different.

The 14 g hook on control branchlines was expected to initially have a relatively slow sink rate up until the point when the 45 g weighted swivel sinks to a depth when the leader becomes taut, and then the weighted swivel increases the hook’s sink rate (Supplementary Fig. S1)^36^. This was why, on the control treatment branchline, we used a sink rate line to attach the bottle to the hook instead of attaching the bottle to the branchline.

TDRs were not used in this study to estimate hook sink rates to 0.85 m because available technology has too low a pressure (depth) resolution to produce accurate estimates over this depth (e.g., the Star-Oddi DST centi-TD has a maximum accuracy of ± 0.3 m, based on ± 0.6% accuracy when using the minimum possible depth range of 50 m)^56^. Furthermore, the mass of the TDR would affect the hook sink rate measurements (e.g., the DST centi-TD TDR with housing and clip weighs 102 g, person communication, Baldur Sigurgeirsson, Star -Oddi, 28 June 2021, however see Ref.^36^).

We did not measure sink rates to deeper depths (e.g., 10 m^26^) using TDRs, or using a bottle test, to estimate the sink rate over the initial 0.85 m because, discussed above, the sink rate of the control branchline, with the weight located ca. 0.75 m from the hook, would not be uniform over this depth profile, where the mean sink rate to 10 m would be faster than to 0.85 m. The use of video reviewing software instead of a hand-held digital stopwatch as used in some previous sink rate studies (stopwatch to measure to nearest 0.01 s^36^) likely produced more accurate estimates given the relatively short intervals involved. Hand-held digital stopwatches suffer from two main sources of error: human reaction time and resolution. The video software program we used eliminated these sources of error in our sink rate estimates.

Similar mechanistic studies of weighted hooks to be used in fisheries in other regions that encounter different seabird species complexes need to account for differences in the depth to which seabirds can access baited hooks. If the fishery being studied overlaps with species of shearwaters and *Procellaria* petrels, which are relatively proficient divers, rapidly attaining maximum depths of 10–20 m and 15–25 m, respectively^22,57^, and if secondary interactions occur^17,25^, then sink rate assessments would need to be conducted to substantially deeper depths than assessed here for the US central North Pacific tuna longline fishery.

Mechanistic study findings are a critically important component for integrating and assessing accumulated types of evidence for a candidate management intervention and for making evidence-based policy decisions^66–68^. They enable answering questions about the mechanisms causing a phenomenon^68^, such as a behavioral response to a bycatch mitigation method. Mechanistic studies can improve the understanding of why an observed response to an intervention, such as a bycatch mitigation method, occurs and can help to identify promising new or modified bycatch mitigation approaches.

### Catch rate effect

There are several possible mechanisms that caused the much lower market species catch rates on experimental branchlines:

- Due to the lack of a swivel in the terminal end, experimental branchlines could have had a higher rate of twisting and tangling during the soak^69^, such as from current shear, and during the haulback, reducing fishing efficiency.
- Weighted circle hooks may be less pliable and be less likely to rotate as the catch pulls and turns away from the leader than conventional circle hooks, reducing the probability that a swallowed hook orients so that the point catches in the corner of the mouth as the eye of the hook slides out of the mouth, as occurs with conventional circle hooks^58^.
- When hooked in the corner of the mouth and thrashing, weighted hooks may have caused relatively more tissue damage than control hooks, allowing the weighted hook to more readily tear out than conventional hooks, resulting in a relatively higher escapement rate on the weighted hooks.
- Weighted hooks may have less movement in the water than conventional hooks, making them less appealing, reducing attack rates.
- The lead on the hook might have reduced attacks due to increased visual detection. Pelagic predators may be able to more readily see the weighted hook and avoid preying on the baited hooks, similar to hypothesized effects on catch risk of using more visible wire leaders as compared to monofilament leaders^59^.
- Slight differences in the dimensions and shape of the two hooks may have affected catchability. For example, the wider hook minimum width of the experimental hook could partially explain the effect on catch rates. The larger the hook, the larger an organism’s mouth dimensions need to be to fit it in its mouth. As a result, for species that tend to be caught by ingesting a baited hook, hooks with a larger minimum width reduce the relative catchability of smaller species and of smaller length classes within a species^43^. The effect of hook minimum width on species-specific catch rates, in particular for species that do not have relatively small mouth sizes, is determined by the length frequency distribution of a species that overlaps with a fishery, the difference between the width of two hooks being compared, and the difference in the hook widths relative to the species’ range of mouth sizes^43^. Because there was a small difference in hook minimum widths of 1.5 mm, this likely had a minimal effect on catch rates.

While this is the first assessment of the effect of a weighted hook on longline catch rates, two previous studies assessed the effect on fish catch rates of locating pelagic longline branchline weights adjacent to the hook. A previous randomized controlled experiment also in this U.S. central North Pacific fishery found that locating weighted swivels adjacent to the hook reduced catch rates of retained and discarded species by 54% and 28%, respectively, relative to locating the weight about 75 cm from the hook. The reduced catch rates on experimental branchlines were possibly due to hooks snagging on the swivel and reduced action (movement) due to a narrower hook eye^31^. A study in Australia’s tuna longline fishery found no difference in species-specific fish catch rates between 40 g luminescent LumoLeads at the hook versus 60 g luminescent LumoLeads at 3.5 m from the hook^15^.

### Commercial viability, next steps

The experimental hook caused a substantial reduction in catch rates of marketable species—this large cost to economic viability makes this weighted hook design unsuitable as a seabird bycatch mitigation option. Given the potential of a weighted hook to achieve substantial seabird conservation gains as suggested by the results of the mechanistic sink rate study, as well as the potential for improved crew safety and practicality, and ease of robust compliance monitoring, it is a priority to conduct research on alternative weighted hook designs to overcome the economic costs observed with the experimental hook design used in this study.

Pelagic longline fishers can be reluctant to place weights that are crimped in place close to hooks due to safety concerns. During the haulback, when the branchline is under tension and the catch either severs the branchline or throws the hook, or the line breaks (including when a crimp fails), a weight close to the hook can fly back at the vessel at high velocity as the branchline recoils, which can cause serious injuries and fatalities^19,29,30^. Relocating the weight to the hook eliminates the safety risk from bite-offs—the hook and weight would now be bitten off, while with the conventional design, the weight could fly back at the vessel. However, when catch throws the hook, a weighted hook could pose a larger safety risk to crew than the conventional branchline weighting design—with two objects separated by about 0.75 m flying back at the vessel. But a weighted hook may pass through the water, reducing the recoil velocity relative to a weight attached away from the hook (however, see Ref.^31^). Eliminating flybacks when bite-offs occur likely achieves a larger reduction in safety risk than potential increased risk from flybacks with a weighted hook that occur when catch throw the hook. Flybacks that occur when catch throw the hook are a risk for all branchline weighting designs, both designs with weight crimped in place and with sliding weights^29–31^.

As with conventional branchline weights that are crimped into the line, weighted hooks facilitate a broader range of approaches for robust compliance monitoring relative to sliding weights and hook shielding devices^29–31,60,61^. While the presence of weighed hooks can be determined through dockside monitoring, crew may decide to move sliding weights further from the hook during a fishing trip, and crew may not attach hook shielding devices to hooks during setting^31^. Thus, sliding weights and hook shielding devices require onboard monitoring to assess compliance.

A weighted hook may cost less than a conventional hook and branchline weight. Reduced bait loss to seabirds would increase fishing efficiency. However, there would be increased costs due to the need to replace a weighted hook instead of only a conventional hook when terminal tackle is lost from bite-offs and when branchlines break or are cut by crew to release catch.

A weighted hook would increase the terminal tackle attached to escaped wildlife from bite-offs and catch that crew release alive by cutting branchlines. Using corrodible hooks or corrodible material for the added weight to the hook may reduce this impact. Using a non-hazardous material instead of lead as used in the prototype weighted hook in this study would minimize adverse ecological effects of derelict gear, including from sublethal and lethal effects from ingestion during terminal tackle bite-offs, and safety risks to crew from handling the gear^62–64^. For example, a weighted hook being designed for trials in Australia and New Zealand will use a single mold so that the hook and weight will both be made of steel^65^.

The study demonstrated the benefits to evidence-based policy decision-making of first conducting mechanistic studies and assessing commercial viability of a prototype bycatch reduction device to determine if a full experiment to assess bycatch mitigation efficacy is warranted. Alternative materials for the hook to increase the mass without increasing hook dimensions, locating the added weight at a different location on the hook, and distributing the added material along a larger proportion of the length of the hook wire warrant exploration. The use of frozen bait infused with non-toxic weight^31^ could be a commercially viable alternative. Testing the weighted hook with a swivel incorporated into the terminal end of the branchline may improve catch rates. The findings of the mechanistic sink rate experiment suggest that weighted hooks have a high potential for seabird conservation gains. Experiments in fisheries with different seabird species complexes are a priority to test this. Weighted hooks could also eliminate crew safety risks from flybacks due to bite-offs, facilitate compliance monitoring without onboard observer or electronic monitoring coverage, and could potentially reduce gear costs. Therefore, trials of alternative weighted hook designs that may result in acceptable catch rates are warranted.

## Supplementary Information

Supplementary Information.

## Data Availability

The datasets generated during and/or analysed during the current study are available from the corresponding author on reasonable request.
