# Supplementary material for: Investigating weighted fishing hooks for seabird bycatch mitigation
Source: Sci Rep. 2022 Feb 18;12:2833. doi: 10.1038/s41598-022-06875-4 (PMC8857297; doi:10.1038/s41598-022-06875-4)
Supplement: Supplementary file 1 — Supplementary Information. [file 41598_2022_6875_MOESM1_ESM.pdf]

**Supplemental Material**

# Investigating weighted fishing hooks for seabird bycatch mitigation

Eric Gilman, Michael Musyl, Michael Wild, Hua Rong, Milani Chaloupka

## Sink Rate Statistical Modeling Approach and Results

The Gaussian GLMM was implemented using weakly informative regularizing priors (Lemoine 2019, Ott et al 2021). The random effect structures (intercepts-only) included in the GLMM was the identity of the 51 unique trial pair to account for any trail-specific heterogeneity or correlation not accounted for by the model. Model convergence was assessed using parameter-specific diagnostics such as multiple chain rank plots, bulk and tail effective sample size metrics and a rank-based *Rhat* statistic (Vehtari et al 2021). Further evaluation of the model fit was assessed using graphical posterior predictive checks (Gelman et al 2014, Gabry et al 2019). The posterior samples for the fitted GLMM were sourced from 4 chains and 2000 iterations after a warmup of 1000 iterations per chain. The posterior for each treatment-specific estimate comprised 4000 samples or draws, where treatment is a 2-level factor comprising experimental or control hooks. Those draws were then summarized as the posterior mean and highest posterior density intervals (HDIs) (Kruschke & Liddell 2018) using both the `tidybayes` package for R (Kay 2020a) and the stat_halfeye() function from the `ggdist` package for R (Kay 2020b). This summary display, showing both the full posterior distribution of each parameter, coupled with 3 posterior summary metrics (mean, 80% HDI, 95% HDI), which supports a precise form of communicating parameter uncertainty (van der Bles et al. 2019). The posterior ratio (and 95% HDI) based on 4000 posterior draws for the 2 densities was then used to assess any apparent difference between the 2 hook treatments (control, experimental) The posterior ratio summary was also included in the expected hook treatment effect summary plot. The `ggplot2` (Wickham 2016) and `colorspace` (Zeileis et al 2020) packages for R were used for the summary graphics.

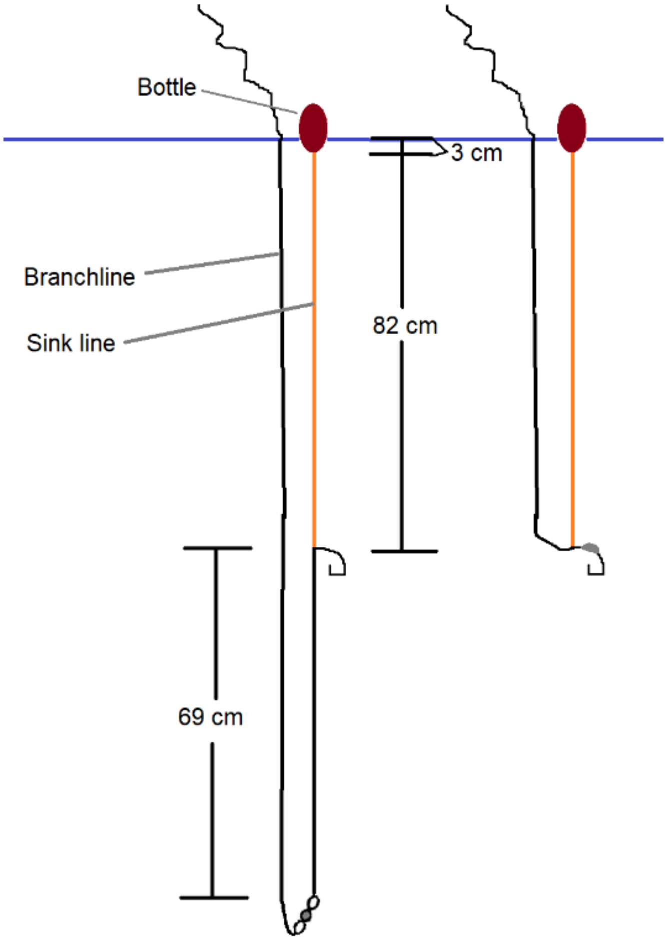


**Figure S1**. Bottle test design to estimate elapsed time for a control hook on a conventionally-designed branchline with a 45 g swivel located 69 cm from the hook (left) and an experimental weighted hook on a branchline without a weighted swivel (right) to sink to 85 cm, showing the position of branchlines once hooks have reached 85 cm.

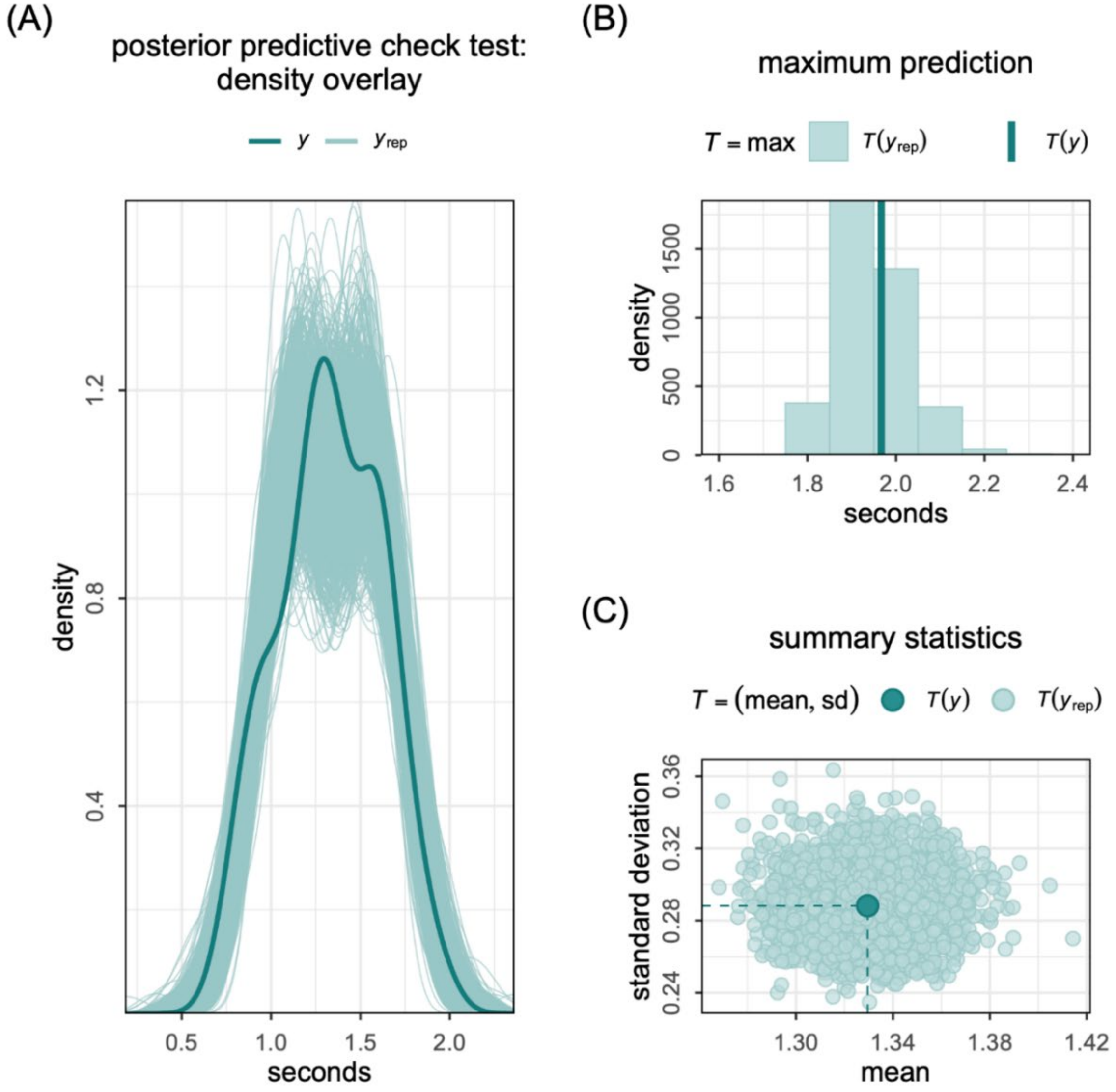


**Figure S2**. Posterior predictive check tests for 1000 randomly selected draws from the GLMM with Gaussian likelihood fitted to the sink elapsed time data. Panel A shows the posterior predictive check for the response variable (time to sink to 85 cm depth) where the solid curve (y) is the density curve summarizing the observed sink data while the mass of curves (*yrep*) are 1000 model-based simulations of the expected sink elapsed time. Panel B shows check for the maximum sink elapsed time where the solid green line shows the maximum of the observed data, and the light green histogram summarizes 1000 simulations of the expected or predicted maximum for the fitted model. Panel C shows a check for 2 key summary parameters (mean and standard deviation of the observed sink data) where the solid dot = observed bivariate estimate and the mass of dots (T(*yrep*)) are the bivariate estimates for the 1000 model-based simulations. All check tests show that the model was an adequate fit to these data and therefore appropriate for inference.

**REFERENCES**

Gabry J, Simpson D, Vehtari A, Betancourt M, Gelman A (2019) Visualization in Bayesian workflow. Journal of the Royal Society Series A 182 (1): 1-14

Gelman A, Hwang J, Vehtari A (2014) Understanding predictive information criteria for Bayesian models. Statistics and Computing 24: 997–1016

Kay M (2020a) tidybayes: tidy data and geoms for Bayesian models. R package version 2.1.1. doi: 10.5281/zenodo.1308151

Kay M (2020b) ggdist: Visualizations of distributions and uncertainty. R package version 2.3.0, URL: https://mjskay.github.io/ggdist/
Kruschke J, Liddell T (2018) The Bayesian New Statistics: Hypothesis testing, estimation, meta-analysis, and power analysis from a Bayesian perspective. Psychonomic Bulletin & Review 25: 178–206
Lemoine N (2019) Moving beyond noninformative priors: why and how to choose weakly informative priors in Bayesian analyses. Oikos 128: 912-928
Ott M, Plummer M, Roos M (2021) How vague is vague? How informative is informative? Reference analysis for Bayesian meta-analysis. Statistics in Medicine 40: 4505–4521
van der Bles A, van der Linden S, Freeman A, Mitchell J, Galvao A, Zaval L, Spiegelhalter D (2019) Communicating uncertainty about facts, numbers and science. Royal Society Open Science 6: 181870
Vehtari A, Gelman A, Simpson D, Carpenter B, Bürkner P (2021) Rank-normalization, folding, and localization: an improved Rhat for assessing convergence of MCMC (with Discussion). Bayesian Analysis 16: 667–718
Wickham H (2016) ggplot2: Elegant Graphics for Data Analysis. 2nd Edition. Springer-Verlag, New York, USA
Zeileis A, Fisher J, Hornik K, Ihaka R, McWhite C, Murrell P, Stauffer R, Wilke C (2020) colorspace: A toolbox for manipulating and assessing colors and palettes. Journal of Statistical Software 96: 1-4
